# A pilot study investigating changes in neural processing after mindfulness training in elite athletes

**DOI:** 10.3389/fnbeh.2015.00229

**Published:** 2015-08-27

**Authors:** Lori Haase, April C. May, Maryam Falahpour, Sara Isakovic, Alan N. Simmons, Steven D. Hickman, Thomas T. Liu, Martin P. Paulus

**Affiliations:** ^1^Department of Psychiatry, University of California, San DiegoSan Diego, CA, USA; ^2^Veteran’s Affairs San Diego Healthcare SystemSan Diego, CA, USA; ^3^Center for Functional MRI, Department of Radiology, University of California, San DiegoSan Diego, CA, USA; ^4^Laureate Institute for Brain ResearchTulsa, OK, USA

**Keywords:** mindfulness, fMRI, interoception, insula, athletes, breathing, anterior cingulate cortex

## Abstract

The ability to pay close attention to the present moment can be a crucial factor for performing well in a competitive situation. Training mindfulness is one approach to potentially improve elite athletes’ ability to focus their attention on the present moment. However, virtually nothing is known about whether these types of interventions alter neural systems that are important for optimal performance. This pilot study examined whether an intervention aimed at improving mindfulness [Mindful Performance Enhancement, Awareness and Knowledge (mPEAK)] changes neural activation patterns during an interoceptive challenge. Participants completed a task involving anticipation and experience of loaded breathing during functional magnetic resonance imaging recording. There were five main results following mPEAK training: (1) elite athletes self-reported higher levels of interoceptive awareness and mindfulness and lower levels of alexithymia; (2) greater insula and anterior cingulate cortex (ACC) activation during anticipation and post-breathing load conditions; (3) increased ACC activation during the anticipation condition was associated with increased scores on the describing subscale of the Five Facet Mindfulness Questionnaire; (4) increased insula activation during the post-load condition was associated with decreases in the Toronto Alexithymia Scale identifying feelings subscale; (5) decreased resting state functional connectivity between the PCC and the right medial frontal cortex and the ACC. Taken together, this pilot study suggests that mPEAK training may lead to increased attention to bodily signals and greater neural processing during the anticipation and recovery from interoceptive perturbations. This association between attention to and processing of interoceptive afferents may result in greater adaptation during stressful situations in elite athletes.

## Introduction

The ability to perform well during a high intensity competition is an important characteristic for elite athletes. For instance, during a difficult competition, a successful athlete adopts a proactive style in optimizing his performance. In contrast, a less successful individual may adopt a simple recovery from insult where competition difficulties cause a period of panic or fear of future failure without an attempt to modify habitual coping mechanisms. There has been mounting evidence to suggest that mindfulness training may improve the brain’s response to stressful situations (McNabb et al., 2012; Armananzas et al., 2013; Austin et al., 2013; Haase et al., 2014). Mindfulness is “the awareness that emerges through paying attention on purpose, in the present moment, and non-judgmentally to the unfolding of experience moment by moment" (VanderWeele and Robins, 2010). We have recently published two studies examining the effect of mindfulness training in active duty infantry marines (Haase et al., 2014; Johnson et al., 2014). Marines who underwent an 8-week mindfulness training program demonstrated (1) attenuated brain response in the right insula and anterior cingulate cortex (ACC) during an emotion processing task (Johnson et al., 2014); (2) attenuated right insula and anterior cingulate activation in response to a physiological probe (Haase et al., 2014); and (3) a positive correlation between attenuated right anterior insula (AI) activation and increased self-reported resilience (Johnson et al., 2014).

Interoception consists of the receiving, processing, and integration of body-relevant signals together with external stimuli to affect motivated behavior (Craig, 2002, 2009). Interoceptive processing is critical for optimal performance because it links disturbances in the body’s internal state caused by external stimuli to goal-driven behaviors aimed at restoring homeostatic balance (Paulus et al., 2009). Moreover, there is increasing evidence that interoceptive processing is a critical component to mindfulness (Farb et al., 2015). We previously proposed that producing body prediction errors, i.e., evaluating the difference between an anticipated/predicted interoceptive state and the actual interoceptive state experienced in response to significant perturbations, might be a neural marker of optimal performance (Malley et al., 2012). This notion is consistent with findings that elite athletes are acutely aware of bodily signals (Philippe and Seiler, 2005) and may more readily produce anticipatory prediction errors (Aglioti et al., 2008). This suggests that modifying interoception may be an experimental target for improving an individual’s ability to respond to internal perturbations brought about by external stressors. However, further research is needed to determine if resilience, a critical characteristic of optimal performance in extreme environments, has a significant influence on brain structures thought to be important for such performance.

In the present investigation, an inspiratory breathing load task is used as the experimental interoceptive provocation probe. Loaded inspiratory breathing was first introduced in the 1970s (Lopata et al., 1977; Gottfried et al., 1978) as an effective task that can produce changes in experimental breathing. Breathing is a vital human function and any interference with breathing can produce aversive affective experiences (Pappens et al., 2010), that provide information about potential threats, leading to increased anxiety (von Leupoldt et al., 2011). The perceived magnitude of an inspiratory load is a function of the inspiratory pressure and indirect function of the added resistance (Killian et al., 1982). Experientially, loaded inspiratory breathing has a sensory component of increased work to inhale but also an affective component that ranges from mild discomfort to intense fear of the inability to breathe in. These characteristics make breathing an ideal experimental probe for the interoceptive system, in particular the insular cortex, as well as a robust interoceptive stimulus.

Previous neuroimaging literature has identified several networks within the brain including medial default mode network, a frontal control network, and a limbic salience network (Spreng et al., 2013). Various approaches have been developed to characterize the function of the insular cortex. One such approach divides the insular cortex into two (Taylor et al., 2008) or three (Deen et al., 2011) compartments, with each compartment serving a different function in these large scale networks. The dorso-AI is commonly associated with the frontal control network (Chang et al., 2012), but other findings suggest that the AI is critical for the saliency network and is functionally connected with frontal, cingulate, parietal, cerebellar brain areas (Sullivan et al., 2013). The posterior insula is closely connected to sensorimotor, temporal and posterior cingulate areas (Cauda et al., 2012). In addition, it has been proposed that the right fronto-insular cortex, in conjunction with ACC, plays a causal role in alternating between the frontal control network and the default mode network (Prosperi et al., 2009) and is involved in switching during a variety of perceptual, memory, and problem solving tasks (Schulz et al., 2015). Consistent with this notion is the observation that the AI is involved in the processing of temporal predictions (Limongi et al., 2013) as well as the influence of self-regulation on functional connectivity (FC; Haller et al., 2013). These connectivity patterns suggest that the AI is important for translating emotional salience into activation of the cognitive control network to implement goal-directed behavior (Cloutman et al., 2012). Interestingly, the insula has significant downstream influence on the nucleus accumbens and striatum, brain areas that are central for reward-related processing (Cho et al., 2012). Taken together, the insular cortex is likely to be a temporally predictive switching structure to serve large neural networks to engage in motivated behavior. Here we focused on the FC with posterior cingulate cortex (PCC), which was found to have a role in self-related and self-referential aspects of cognitive processing (Whitfield-Gabrieli and Ford, 2012; Garrison et al., 2013; Brewer and Garrison, 2014). Lower connectivity between PCC and ACC during a task was found in meditators when compared to novices (Brewer and Garrison, 2014), which points to the role of these regions in self-related processings. In the present study, we are interested in determining if a manualized treatment, Mindful Performance Enhancement, Awareness and Knowledge (mPEAK), can produce similar changes in FC as those seen in experienced meditators.

The primary aims of this pilot study are to examine if a 7-week (2 full-day sessions and 6 weekly, 90 min sessions) intervention aimed at improving mindfulness by targeting regions identified in previous neuroimaging studies can modify how the brains of elite athletes process interoceptive stimuli; FC in the default mode network during a resting state scan; and if this change in challenging situations is a function of the insula and ACC and if these regions are modulated by mindfulness training. Furthermore, if predicting perturbations in the internal body state is a function of AI and ACC, we hypothesize that heightened activation in these structures is associated with increased resilience.

## Materials and Methods

### Participants

The study was conducted at the University of California, San Diego (UCSD) Center for Functional MRI. The UCSD Institutional Review Board approved this study and all participants signed informed consents. Participants were recruited from the USA BMX (Bicycle Motocross) Cycling Team (*n* = 7) and underwent a 7-week mindfulness training program (see **Table 1** for intervention outline). See **Table 2** for demographic information.

**Table 1:** Mindful Performance Enhancement, Awareness, and Knowledge.

**Session 1**	**Module 1: Inhabiting your body (180 min)**
	• The body as the primary focus of attention
	• Mindful awareness of the body; body scan
	• Research on interoception, optimal performance, and mindfulness
	• Experiential Exercise: straw breathing
	• Home practice assignment
**Session 1**	**Module 2: Getting out of your own way and letting go (180 min)**
	• The wandering mind and recognizing how “story” influences performance
	• Mindful movement; seated meditation
	• Research on the default mode network
	• Experiential exercise: Efforting versus letting go
	• Home practice assignment
**Session 2**	**Module 3: Working with difficulty (180 min)**
	• Confronting avoidance in the face of difficulty, by working with the body
	• Seated meditation focusing on difficulty; seated meditation focusing on letting go
	• Research on pain and negative affect
	• Experiential exercise: ice bucket
	• Home practice assignment
**Session 2**	**Module 4: The pitfalls of perfectionism and the glitch in goals (180 min)**
	• Identification of strengths and their “dark side”
	• Mindful walking, seated meditation
	• Research on perfectionism and self-criticism
	• Experiential exercise: Compassionate inner coach
	• Home practice assignment
**Session 3–8**	**Six, weekly, foundational Practice Sessions (90 min)**
	• Check-in
	• Supporting practice through injury and discussion
	• Mindfulness practice

**Table 2:** Demographics and self-report measures of study participants.

	mPEAK (*n* = 7)		

	***M* (SD)**		
**Demographics**			
Age	21.86(3.67)		
Education (years)	12.57(0.98)		

	**mPEAK T1 *M* (SD)**	**mPEAK T2 *M* (SD)**	***p***

**MAIA**			
Noticing	3.92 (0.26)	3.67 (0.74)	
Not-distracting	2.19 (1.17)	2.48 (1.15)	
Not-worrying	2.67 (0.82)	3.00 (0.54)	
Attention regulation	2.97 (0.70)	3.43 (0.49)	
Emotional awareness	3.91 (0.77)	4.06 (0.19)	
Self-regulation	2.86 (0.85)	3.61 (0.64)	0.009
Body listening	2.00 (1.45)	2.71 (0.78)	
Trusting	3.71 (0.56)	4.42 (0.37)	0.008
**TAS**			
Describing feelings	9.57(4.20)	8.57 (4.61)	
Identifying feelings	12.71 (5.25)	8.43 (1.40)	0.037
External thinking	13.57 (2.15)	14.57 (4.65)	
**FFMQ**			
Describe	25.29 (6.99)	30.43 (5.16)	0.007
Acting with awareness	27.29 (4.54)	26.29 (4.86)	
Non-judgment	23.71 (7.34)	24.42 (5.74)	
Non-reactivity	19.29 (4.61)	22.85 (3.02)	
Observation	26.29 (3.20)	28.57 (6.34)	
**VAS ratings**			
Pleasantness	3.32 (2.83)	5.74 (3.77)	
Unpleasantness	2.80 (2.44)	1.91 (2.86)	
Intensity	1.04 (1.81)	0.37 (0.51)	
**Response latency (msec)**			
Baseline	597 (101)	603 (142)	
Anticipation	628 (134)	684 (171)	
Load	580 (113)	595 (160)	
Post-load	770 (147)	842 (169)	
**Accuracy (%)**			
Baseline	98 (2)	95 (8)	
Anticipation	99 (1)	95 (6)	
Load	98 (1)	95 (10)	
Post-load	100 (0)	94 (10)	

### Study Design

The BMX group underwent two fMRI scans: (1) baseline assessment, which occurred 3 days prior to the mPEAK course and (2) post-training, which occurred approximately 1 week following the mPEAK course.

### Intervention

Mindful Performance Enhancement, Awareness and Knowledge is a 7-week intensive course in mindfulness training that was built around four core modules (2 modules per day, 3 h per module, over two consecutive days) with 6 weekly follow-up foundational practice sessions (90 min per session) to solidify and deepen the practice and skills being taught in the core modules. See **Table 1** for an outline of the program. The practice sessions support the ongoing integration of mindfulness practice into daily routines in the workplace, the training environment or in competition. All sessions provide a forum for dialog, questions and comments. The foundation of this program was drawn from the highly respected and empirically supported Mindfulness-Based Stress Reduction program (Sugimoto et al., 2013). The first core module establishes the body as the primary focus of attention and platform upon which mindfulness unfolds. It is designed to introduce participants to the experience of mindful awareness of the body, including interoception and proprioception. The second core module is devoted to addressing initial challenges encountered by participants, the universality of the wandering mind and the fruitlessness of trying to stop the mind from wandering. Recognizing “story” and how the way in which we think influences our performance. The third core module is intended to challenge the notion that avoidance is the best strategy when it comes to difficulty arising (e.g., pain, fear, stress, failure, etc.) and to use the experience of working with the body as a way of grounding oneself in the moment in the face of difficulty. The fourth and final core module deals specifically with the contradictory nature of some concepts and attitudes that seem to be positive, but have some hidden limitations. Perfectionism and self-criticism can seem to be good motivators, but research clearly shows that people perform more effectively when motivated by encouragement, reward and self-compassion. Specific exercises and practices are taught to address these findings and support people in finding optimal ways to motivate themselves and achieve their goals. A secondary intention of this module is to set the stage for continued regular personal practice of mindfulness through the post-intensive period. The four core modules are followed by 6 weekly foundational practice sessions. These sessions are dedicated to checking in with participants, supporting their ongoing practice through inquiry, discussion and mindfulness practice. Each session also includes a specific relevant topic to focus the meeting and reinforce the importance of continued practice. Participants were encouraged to practice mindfulness and self-regulation skills daily, for at least 30 min.

### Self-Report Assessments

The BMX participants completed several self-report questionnaires to assess personality and cognition pre- and post-fMRI scans. Questionnaires that were administered at baseline and post-training included: (1) Five Facets of Mindfulness Questionnaire (FFMQ; Haxby, 2012), designed to measure the five primary facets of mindfulness (observing, describing, acting with awareness, non-judging of inner experience, and non-reactivity to inner experience); (2) Multidimensional Assessment of Interoceptive Awareness (MAIA; Lanza et al., 2013) designed to measure body awareness and responsiveness; and (3) Toronto Alexithymia Scale (TAS; Bagby et al., 1994) designed to measure the ability to identify and describe one’s emotions.

### Subjective Interoceptive Assessment

Participants wore a nose clip and respired through a mouthpiece with a non-rebreathing valve (2600 series, Hans Rudolph) that maintained an airtight seal. The resistance loads consisted of a stainless steel mesh screen within a Plexiglas tube (loading manifold), Before the scanning session, participants experienced a 1 min breathing restriction (40 cmH_2_O/L/sec) practice run and rated the experience. Using a 10 cm Visual Analog Scale, participants provided ratings of the pleasantness, unpleasantness and intensity of the breathing restriction ranging from “not at all” to “extremely.”

### Functional MRI Inspiratory Breathing Load (IBL Task)

The basic experimental approach is analogous to the behavioral interoceptive task described above. Inside the scanner, the mouthpiece was positioned comfortably between the lips and was attached to the scanner head coil to eliminate the need for the participant to contract mouth muscles. During the scanning session, a simple continuous performance task was administered. For the task, a single black arrow was presented one at a time overlaid on a colored rectangle and participants pressed one of two buttons to indicate the direction of the arrow (left arrow = left button, right arrow = right button). At the same time, the color of the rectangle served as a cue to the likelihood of whether the participant would experience the breathing load in the next set of trials (blue = no load, yellow = 25% chance of load). The 25% probability was introduced in order to maximize the opportunity to measure the effects of uncertain anticipation of an interoceptive stimulus. Accuracy and response latency were recorded and analyzed to determine effects of the anticipation and experience of the breathing restriction. Randomly varied inter-trial intervals were used between each anticipation phase. There were four conditions (1) baseline: the rectangle color signaled that there was no chance of experiencing the breathing load; (2) anticipation: the color of the rectangle signaled a 25% probability of experiencing the breathing load for 40 s; (3) breathing load: the rectangle remained yellow for 40 s while the participant experienced restricted breathing; and (4) post-breathing load: immediately following the 40 s period of restricted breathing. Subjects were instructed to maintain a consistent breathing pace during the scan and exhaled CO_2_ was measured. This paradigm used an event-related design and total scan duration was 17 min and 4 s. The paradigm was divided into two runs of 256 repetitions each (2 s each repetition). The duration of each condition was “jittered” in time to maximize the resolution of the hemodynamic response function. The primary behavioral variables were performance accuracy and response latency during each condition, and the primary neuroimaging dependent measure was the activation in functionally constrained regions of interest during the anticipation and breathing load condition relative to the baseline condition (for additional task-related details see Paulus et al., 2012).

### Scanning Parameters

Imaging data were acquired at the UCSD Center for Functional MRI on a 3T GE MR750 scanner, equipped with an eight-channel high bandwidth receiver. A high-resolution anatomical image was obtained, which consisted of a sagittally acquired spoiled gradient recalled (SPGR) sequence (172 sagittal slices; FOV 25 cm; matrix: 192 × 256 (interpolated to 256 × 256); slices thickness: 1 mm; TR: 8 ms; TE: 3 ms; flip angle: 12°). A standard gradient echo-planar images (EPI) pulse sequence was used to acquire T2^∗^-weighted functional images (40 axial slices, FOV: 230 mm, matrix: 64 × 64; slice thickness: 3 mm; TR: 2000 ms; TE 32 ms; flip angle: 90°). Rapid image T2^∗^ acquisition was obtained via GE’s ASSET scanning, a form of sensitivity encoding (SENSE), which uses parallel imaging reconstruction to allow for sub k-space sampling.

### Image Analysis Pathway

All participant-level data were processed with the Analysis of Functional NeuroImages (AFNI) software package (Cox, 1996), including temporal and spatial alignment, motion and outlier detection, concatenation, deconvolution, and Talairach transformation. Orthogonal regressors were computed for four conditions: (1) baseline (2) anticipation, (3) breathing load, and (4) post-breathing interval. A task-based reference function corresponding to the time interval of the regressor of interest was convolved with a gamma variate function (Boynton et al., 1996) that modeled the prototypical 6–8 s delayed hemodynamic response function (Friston, 1995) and the temporal dynamics of the hemodynamic response (typically 12–16 s; Cohen, 1997). For each participant, three motion parameters were acquired and used to adjust for any EPI intensity changes resulting from motion artifacts. To be excluded, the average of any one of these parameters had to exceed 2 SD from the mean or movement had to be greater than the size of the voxel (4 mm); however, no participant was excluded based on this criterion. Using the AFNI program, 3dDeconvolve, multivariate regressor analysis was used to relate changes in EPI intensity to differences in task characteristics. The main dependent measure was percent signal change, which was spatially smoothed with a 4 mm full-width half-maximum Gaussian filter.

### Group Level Analysis

The main dependent measure was percent signal change during the anticipation, breathing load and post-breathing load conditions, which were entered into a mixed effects model (Littell et al., 2000). Linear mixed effects models were conducted in R (http://cran.r-project.org/), which estimates parameters using Maximum Likelihood Estimation and estimates effects using specific contrast matrices. Time (baseline versus post-training) was included as a fixed factor while subject was entered as a random factor, and a covariate of baseline activation was included. Each experimental condition (anticipation, stimulation, and post-stimulus intervals) was analyzed separately. The AFNI AlphaSim program estimates statistical significance based on Monte-Carlo simulations and as such was employed to calculate voxel-wise statistics and protect against Type I errors. Given the spatial smoothing of 4 mm FWHM and a voxel-wise *p* < 0.05, the volume threshold for cluster-wise probability of 0.05 for the whole brain analysis was determined to be 768 uL using the AlphaSim program, the equivalent per voxel uncorrected threshold is *p* = 0.00002193. To be considered for further analysis, clusters were required to meet these criteria.

### Resting State Data Analysis and Functional Connectivity

Ten minutes of fMRI resting state data with eyes open (with fixation) were acquired for each subject. Resting state functional data were corrected for time-shift, motion, and field inhomogenities, then transferred to standard space, and resampled to 3 mm^3^ isotropic voxels. Nuisance regressors removed from the resting data included: (1) linear and quadratic trends, (2) six motion parameters and their first derivatives, and (3) mean WM and CSF signals and their first derivatives. Each functional volume was spatially smoothed to 6mm FWHM and low pass filtered with a cut-off frequency of 0.1 Hz. FC analysis was then performed on the data. The seed region of interest (ROI) chosen for FC was a 6 mm-radius sphere in the PCC with the coordinates described in (Van Dijk et al., 2010). Connectivity maps were generated by computing the correlation between the average time series in the PCC and all other voxels in the brain. Correlation maps were subsequently normalized to z-scores using the Fisher-Z transformation. Paired *t*-tests were used to compare the pre- and post-training FC. Regions with significant differences (*p* < 0.05) were identified and corrected for multiple comparisons using AlphaSim in AFNI (cluster size = 146 voxels).

### Behavioral Data Analysis

All behavioral data analyses were carried out with SPSS 22.0 (IBM, Chicago, IL, USA). Repeated measures ANOVA (RM-ANOVA) were run to examine differences across time (baseline versus post-mPEAK), separately, for FFMQ, MAIA, and TAS. Additionally, RM-ANOVA were conducted to investigate differences across time (baseline versus post-mPEAK), separately for percent change from baseline for accuracy and response latency during the IBL task (baseline, anticipation, breathing load, and post-breathing load), and for VAS ratings obtained during the behavioral IBL task prior to the scan session. Given that this is a pilot study, with a small sample size, corrections for multiple comparisons were not implemented; results were considered significant at p < 0.05.

### Exploratory Brain-Behavior Correlations

Exploratory correlations were performed between self-report measures and fMRI brain response to anticipation, breathing load and post-breathing load conditions. Correlations were limited to self-report measures that were significantly different from pre- to post-mPEAK and significant fMRI activation in the ACC and insula. Spearman’s Rho (ρ) was used to determine significance of the relationship between the change in self-report measures (e.g., post-mPEAK TAS Identifying Feelings minus pre-mPEAK TAS Identifying Feelings) and the change in fMRI activation (e.g., post-mPEAK ACC activation minus pre-mPEAK ACC activation). Results were considered significant at *p* < 0.05; without corrections for multiple comparisons.

## Results

### Self-Report

The TAS Identifying Feelings subscale changed significantly across time [*F*(1,6) = 7.18, *p* = 0.037, Cohen’s *d* = 1.12], such that, following the mPEAK training, the BMX athletes reported significantly less difficulty identifying feelings (**Table 2**). There was also a significant effect of time for two subscales of the MAIA: self-regulation [*F*(1,6) = 15.54, *p* = 0.009; *Cohen’s d* = 1.51] and trusting [*F*(1,6) = 15.00, *p* = 0.008; *d* = 1.00], such that following mPEAK training, the BMX athletes self-reported greater levels of self-regulation and trust. There was a main effect of time for the FFMQ describe subscale [*F*(1,6) = 15.68, *p* = 0.007; *d* = 0.84], such that following mPEAK training, the BMX athletes self-reported greater levels of describing/labeling with words.

### Subjective Breathing Load and Behavioral Performance During fMRI Breathing Load

There was an overall effect of time for the VAS 40 cmH2O/L/sec pleasantness ratings [*F*(1,6) = 6.91, *p* = 0.039; *d* = 0.73]; pleasantness ratings increased from pre- to post-fMRI sessions (**Table 2**). There was no effect of time for VAS unpleasantness or intensity ratings. For response latency, there was no main effect of time. However, there was a main effect of condition [*F*(3,12) = 20.27, *p* = 0.003]; the response latencies change from baseline were significantly different for all three conditions. For response accuracy, there was no main effect of time.

### Neuroimaging Results

#### Anticipation Main Effect of Time

**FIGURE 1:**
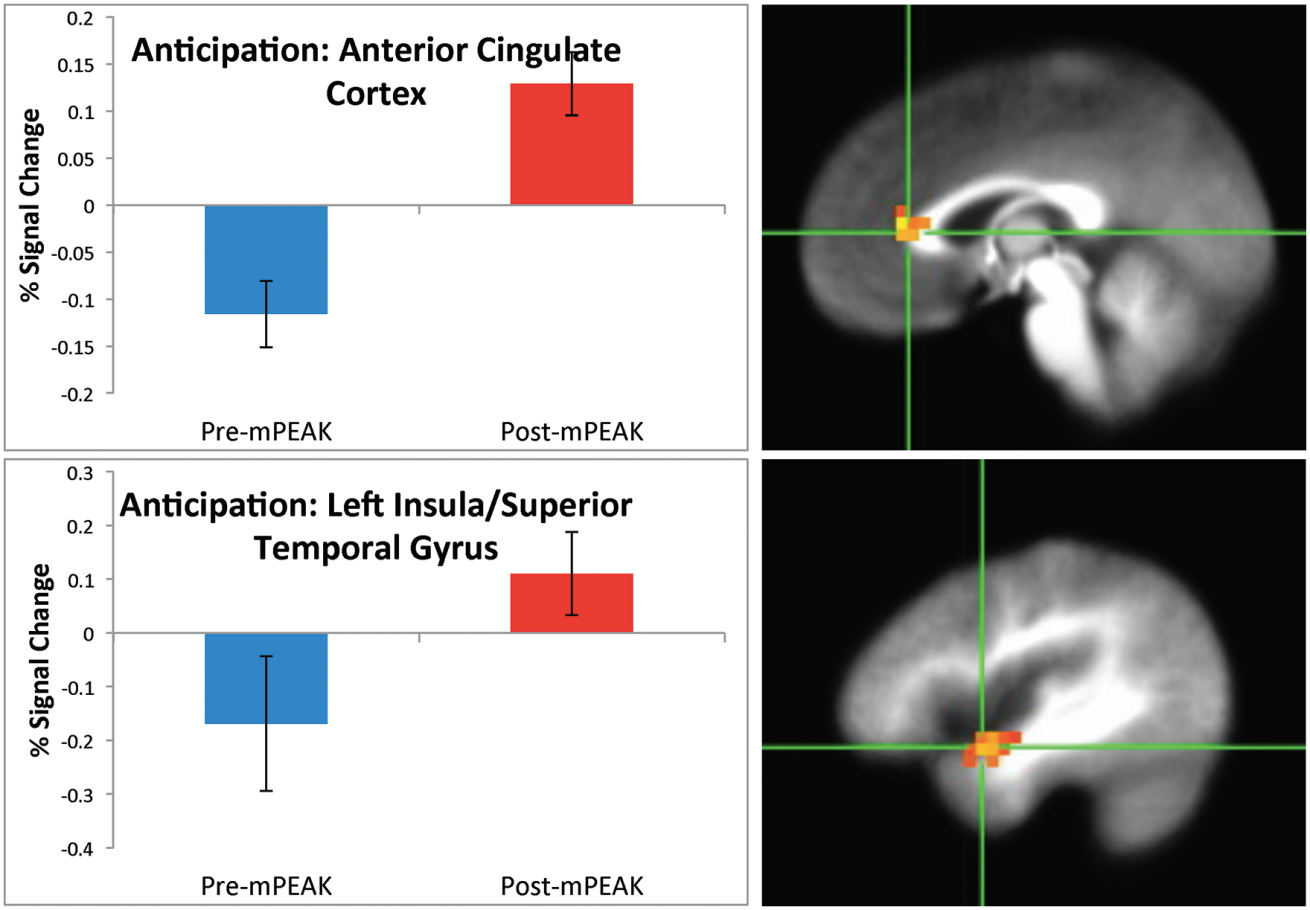
**Percent signal change during the anticipation condition; corrected for multiple comparisons at *p* < 0.05**.

There was a significant effect of time for the anticipation condition in the right cuneus, right ACC (**Figure 1**) left insula and left superior temporal gyrus (**Figure 1**; see **Table 3** for coordinates and effect sizes). For all regions, there was a significant increase in activation following the mPEAK program.

**Table 3:** Functional magnetic resonance imaging (fMRI) brain response during inspiratory breathing load (IBL).

Region	BA	Voxels	*x*	*y*	*z*	*T*-value	*p*-value	*Cohen’s d*
**Anticipation**							
Right cuneus	17	21	10	-91	5	6.264	0.000	5.114
Right anterior cingulate cortex	24	21	4	28	14	6.196	0.000	5.059
Left insula and auperior temporal gyrus	13	13	-42	0	-11	4.029	0.002	3.289
**Breathing load**							
Left lingual gyrus	19	23	-16	-52	1	4.353	0.001	3.554
Right culmen	37	20	43	-45	-19	-5.326	0.000	-4.349
Right caudate		13	18	-18	20	-3.751	0.003	-3.062
Left superior frontal gyrus	6	13	-4	3	61	-5.074	0.000	-4.143
Left supramarginal gyrus	40	12	-62	-43	26	-8.418	0.000	-6.873
**Post-breathing load**							
Left precuneus	31	71	-10	-63	21	-8.230	0.000	-6.719
Left cuneus	18	17	0	-84	15	-4.526	0.001	-3.696
Right dorsolateral Prefrontal cortex	9	14	23	32	32	3.410	0.006	2.784
Right insula	13	12	37	15	-1	3.537	0.005	2.888

#### Breathing Load Main Effect of Time

There was a significant effect of time for the breathing load condition in the left lingual gyrus, right culmen, right caudate, left superior frontal gyrus, and left supramarginal gyrus (see **Table 3** for coordinates and effect sizes). There was a significant increase in activation following the mPEAK program in the left lingual gyrus and a significant decrease in activation in the right culmen, right caudate, left superior frontal gyrus, and left supramarginal gyrus.

#### Post-Breathing Load Main Effect of Time

**FIGURE 2:**
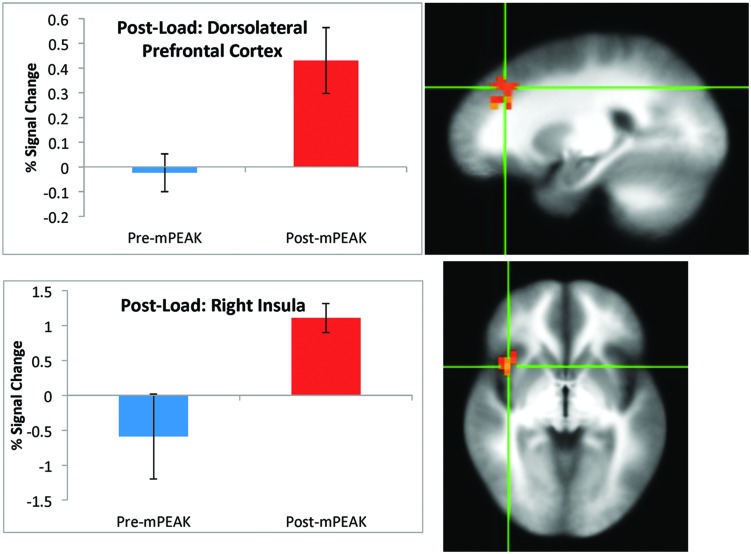
**Percent signal change during the post-breathing load condition; corrected for multiple comparisons at *p* < 0.05**.

There was a significant effect of time for the post-breathing load condition in the left precuneus, left cuneus, right dorsolateral prefrontal cortex (DLPFC; **Figure 2**) and the right insula (**Figure 2**; see **Table 3** for coordinates and effect sizes). There was a significant increase in activation following the mPEAK program in the right DLPFC and right insula, and a significant decrease in activation in the left precuneus and left cuneus.

### Exploratory Brain-Behavior Correlations

**FIGURE 3:**
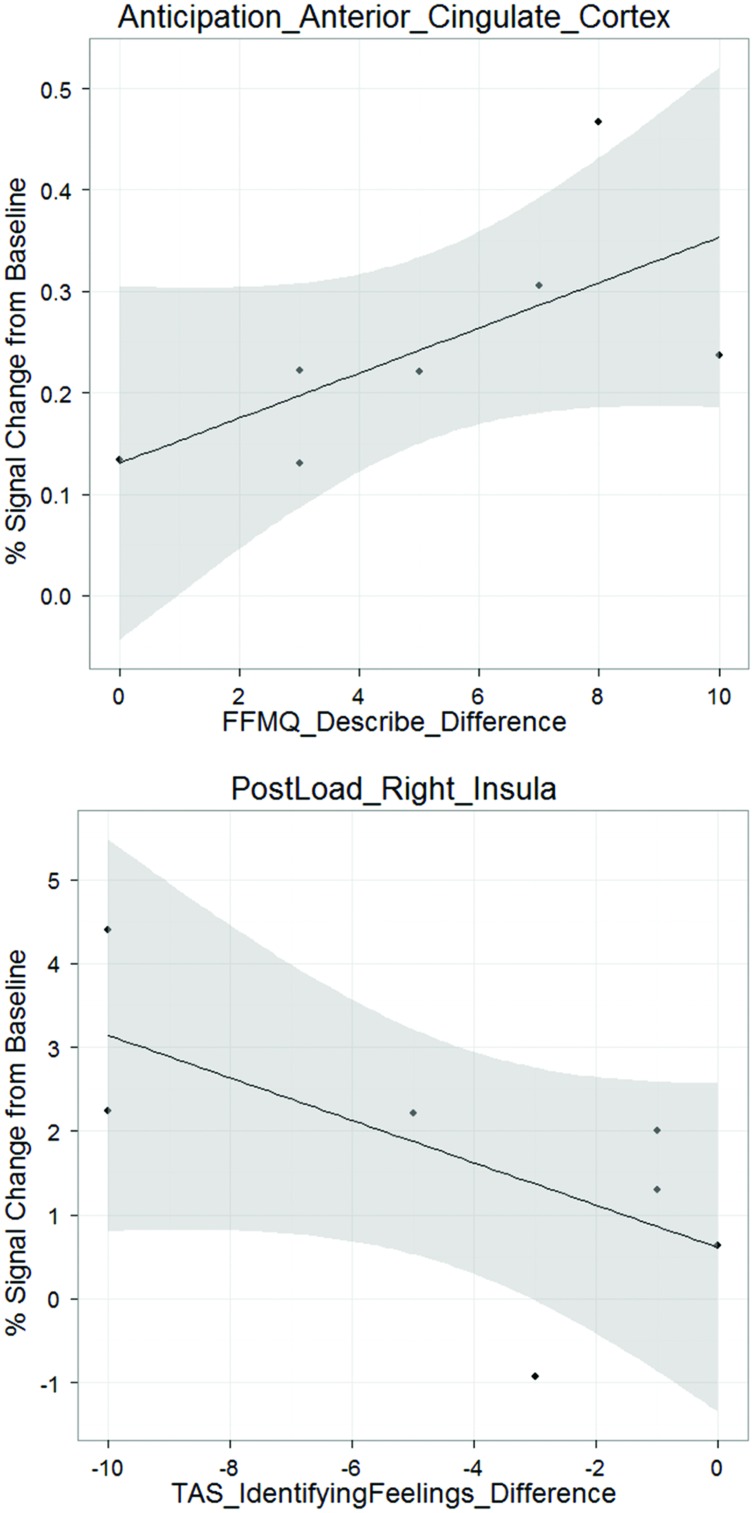
**Exploratory brain-behavior correlations**.

There was a positive correlation between anticipation ACC difference activation and FFMQ *describe difference*, (ρ = 0.775, *p* < 0.05; **Figure 3**). That is, an increase in ACC activation during the anticipation condition from pre- to post-mPEAK is associated with an increase in FFMQ *describe* pre- to post-mPEAK. In addition, there was a negative correlation between post-breathing load right insula difference activation and TAS *identifying feelings difference* (ρ = -0.764, *p* < 0.05; **Figure 3**). An increase in the right insula activation during the breathing load condition from pre- to post-mPEAK is associated with decreased TAS *identifying feelings* – in other words, better ability to describe one’s experience. In summary, greater ability to describe emotions is associated with increased activation in the ACC and right insula, regardless of the self-report measure.

### Functional Connectivity

**FIGURE 4:**
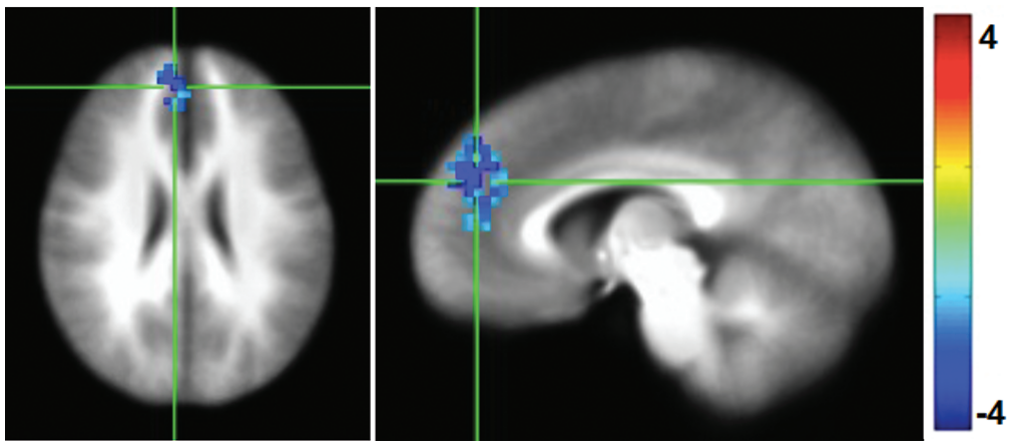
***t*-statistics for changes (Post-mPEAK Minus Pre-mPEAK) in functional connectivity with the seed in PCC (*p*<0.05, corrected); negative values in the figure show the decrease in functional connectivity after mPEAK training**.

Functional connectivity maps were generated by computing the correlation coefficient between the average time series in the PCC and the rest of the brain, and then normalized to Fisher-*Z* values for comparison. Following the mPEAK training there was a significant decrease in the FC with the seed in PCC in one cluster (**Figure 4**). The region contained 152 voxels, with the center of mass at (*x* = -6, *y* = -45, *z* = 25), and primarily included portions of the right medial frontal gyrus, right superior frontal gyrus and right ACC. In other words, this cluster showed lower correlations with the PCC after the mPEAK training.

## Discussion

The present pilot study aimed to examine whether a 7-week (2 full-day sessions and 6 weekly, 90 min sessions) intervention intended to improve mindfulness (mPEAK) modifies neural activation patterns in elite athletes processing interoceptive stimuli and if this change is related to improved self-assessment, and changes in resting state FC. This investigation yielded several interesting findings. First, following mPEAK training, elite athletes self-reported greater levels of interoceptive awareness, mindfulness and lower levels of alexithymia. Second, mPEAK training resulted in increased insula and ACC activation during the anticipation and post-breathing load conditions during an interoceptive challenge. Third, there were significant exploratory brain-behavior correlations, such that increased ACC activation during the anticipation condition following mPEAK training was associated with increased ratings on the FFMQ Describe subscale. Fourth, increased insula activation during the post-load condition following mPEAK training was associated with increased ability to identify feelings on the TAS. Fifth, mPEAK training resulted in decreased PCC FC with the right medial frontal cortex and ACC. These findings are consistent with the hypothesis that mPEAK training resulted in increased attention to bodily signals and greater neural processing in response to the anticipation and recovery from interoceptive perturbations.

Following the mPEAK training, the athletes’ self-reported greater levels of MAIA self-regulation and trusting. MAIA self-regulation refers to the ability to “regulate psychological distress by attention to body sensations” and MAIA trust refers to the ability to “experience one’s body as safe and trustworthy,” both of which are negatively correlated with trait anxiety (Lanza et al., 2013). In addition, the athletes reported greater levels of FFMQ describe and a decrease in TAS difficulty identifying feelings. Taken together, these results suggest that mPEAK training improves one’s ability to identify and describe feelings and one’s reactions to bodily sensations, which may be indicative of reduced trait anxiety and improved resilience.

Mindful Performance Enhancement, Awareness and Knowledge training resulted in changes in functional brain response to an interoceptive challenge. In particular, following the mPEAK training, there was an increase in activation in the left insula during the anticipation of the IBL and in the right insula during the post-breathing load condition. The insula is part of a larger brain network that processes interoceptive information (Wiesner and Pfeifer, 2014). It has reciprocal connections with subcortical, limbic, and executive control brain systems that allow for the integration of interoceptive signals and hedonic evaluation (Marlin et al., 2013; Dasgupta et al., 2014). Several studies have shown increased insula activation during meditation in experienced meditators (Norman et al., 2006; Gopalakrishnan et al., 2010; Yang et al., 2012), suggesting that mindfulness practice, which involves exercises that foster body awareness, recruits interoceptive brain regions. In fact, a recent study by Farb et al. (2015) demonstrated that mindfulness training increased interoceptive awareness of respiratory sensations marked by heightened insular activation (Sohn et al., 2008). We have previously shown that elite adventure racers demonstrate increased anticipatory insula activation during the IBL task and decreased brain activation to the IBL itself (Fernandez-Luque et al., 2009). It was suggested that this pattern of activation in response to an interoceptive stressor may be a neural marker of optimal performance, such that increased engagement of interoceptive brain regions prior to experiencing an interoceptive stimulus may have contributed to a more efficient insula response during the actual experience of an interoceptive challenge (Fernandez-Luque et al., 2009). In summary, this pilot study indicates that mPEAK training results in a general sense of changes in functional activation in neural regions involved in mindfulness meditation and interoceptive processing but as a pilot study the results do not represent anything definitive.

In the present investigation, following mPEAK training, athletes reported on a subjective level (i.e., MAIA), increased interoceptive awareness; however, on an objective level (i.e., VAS ratings and brain activation), the results were mixed. While the athletes demonstrated increased insula and ACC activation during the anticipation and post-load conditions of the interoceptive challenge, their subjective evaluations (pleasantness, unpleasantness, intensity) of the breathing load task did not change. Khalsa et al. (2008) found that experienced meditators rated their heartbeat detection performance to be more accurate and the task to be less difficult than the non-meditators; however, their ability to detect interoceptive changes was not significantly different than non-meditators. Taken together, there may be differences in objective and subjective assessments of interoception, which parallel those of other constructs, such as impulsivity (Wittmann and Paulus, 2008). This also suggests the need for more objective assessments of interoceptive awareness in future investigations, such as the heartbeat detection task (Critchley et al., 2004). In fact, recent research suggests that interoception is comprised of three dissociable dimensions: accuracy, sensibility, and awareness (Garfinkel et al., 2015). Garfinkel et al. (2015) define Future studies aimed at delineating the effects of mindfulness-based meditation on the various facets of interoception are warranted.

This pilot investigation also suggests increased ACC activation during the anticipation of an interoceptive stimulus and increased activation in the DLPFC during the post-breathing load condition. Together, the insula and ACC, appear to have a critical influence on the dynamics between default-mode and executive control networks (Sutherland et al., 2012), integrate information about the internal state with value and salience monitoring systems, and are critical for cognitive control processes. Experienced meditators have exhibited changes in functional ACC activation during attention (Oquendo et al., 2012) and focused meditation paradigms (Baca-Garcia et al., 2006; Zhang et al., 2011; Tovar et al., 2012), and increased cortical thickness (Pasternak et al., 2009). A recent study investigating the effects of mindfulness training on attention found that those who underwent a 6-week mindfulness training program had increased DLPFC functional activation during an affective Stroop task and reduced affective Stroop conflict performance (Bengio, 2007). Furthermore, the authors reported that increased DLPFC, ACC and insula activation during negatively valenced stimuli was related to increased practice time. The authors suggest mindfulness training results in improved top–down control and that the dose-dependent response is in line with other studies (Allefeld and Haynes, 2014) which demonstrate that meditation novices may largely practice focused attention meditation (i.e., awareness of breath), whereas those with a more stable and long-term practice shift from focused attention to open awareness practices.

In prior studies, we have used experimental paradigms that probe emotion processing and interoception to delineate the neural systems underlying optimal performance (e.g., elite athletes, special operations forces). We have identified differential insula and ACC activation during emotion processing and loaded inspiratory breathing (Fernandez-Luque et al., 2009; Van Calster et al., 2010; Zhou et al., 2010; Daemen et al., 2012). Differential activation of insula, ACC and amygdala during emotion processing is also associated with self-reported resilience (Van Gestel et al., 2001; Zhou et al., 2010). The ACC has been conceptualized as the limbic motor cortex (Allman et al., 2001; Craig, 2007), and the critical interface between cognition and emotion processing (Vogt et al., 2003). Taken together, the insula and ACC appear to be important brain regions underlying the ability to perform well under stress. We hypothesize that AI, possibly in combination with the ACC, not only receives interoceptive signals but also produces a predictive model that acts as a signal to the individual of how the body will feel, a concept we have termed the body prediction error. Recently, we have examined the neural and behavioral mechanisms of resilience and have found that individuals who self-report lower levels of resilience show increased activation in the insula, which is negatively associated with mindfulness and interoceptive awareness (Haase et al., under review). In summary, these pilot data provide additional support that mindfulness and interoception are important characteristics of resilience and that neural circuits of resilience can be modified through mindfulness training in relatively healthy, non-treatment seeking individuals.

The resting state FC analysis was aimed to determine whether the mindfulness training would affect the resting state FC in DMN with the focus on its midline node, PCC. Following the mPEAK training we found decreased FC between PCC and right medial frontal gyrus, right superior frontal gyrus and right ACC. A similar result was found during task where meditators showed lower FC between the PCC and other cortical midline structures including the ACC and mPFC (Garrison et al., 2013). PCC activation is thought to be related to multiple aspects of cognitive processes including attention disruption, craving, and self-referential processing while its deactivation is associated with being mindful of the present moment (Brewer et al., 2013). Lower connectivity between PCC and mPFC following mindfulness training may indicate reduced self-referential processing and may facilitate shifting between the frontal control network and the default mode network (Prosperi et al., 2009).

We have previously seen an inverse relationship between anticipatory activation and stimulus related activation during the IBL task. High resilient individuals appear to show relatively greater activation during anticipation (Paulus et al., 2012). Conversely, low resilient individuals have an exaggerated response during the stimulation (Haase et al., under review). Here, increased ACC activation during the anticipation condition was associated with increased FFMQ describe subscale and increased insula activation during the post-breathing load condition was associated with greater ability to identify emotions. Increased anticipatory activation in the ACC following mindfulness training, may improve mindful processing during a time period that is about to come to pass. Additionally, increased insula activation during the post-breathing load may represent the immediate predication of interoceptive relief. Therefore, ultimately mindfulness might not be about the here and now but about ‘what is about to occur.’ Thus, mindfulness may help to prepare the brain for significant perturbations and enable the execution of adaptive responses, which helps to build resilience.

There are several limitations in the current investigation. First, we recruited 7 BMX athletes from the USA Cycle Team. Having such a focused recruitment population, meant that we could not recruit 7 more BMX athletes at this level for a control group. Given the small sample size, generalizability of findings is limited and there may be a lack of power to detect additional behavioral/functional relationships. As a result of sample size limitations, we chose not to control for multiple comparisons in the behavioral data and brain-behavior relationship analyses. As such, these data are prone to Type I error that is, classifying an effect as significant when it is not. A larger sample size might reveal additional significant findings; however, despite the small sample size, significant self-report and functional brain changes were observed. Nonetheless, future studies with larger sample sizes and across various athletic populations are warranted to better understand how mPEAK impacts brain and behavior. Second, this study could not address the question of whether the observed changes were part of the preexisting characteristics of individuals who were selected and then trained to become elite athletes, or whether these neural processing differences were a consequence of training. However, the reported effects are investigating the longitudinal changes within individuals following mPEAK training. Third, this investigation lacked a comparison group and an active control group. However, it should be noted that the present findings are consistent with the literature documenting the effects of mindfulness-based training on ACC and insula functional activation. Furthermore, without a control group, it is possible that the changes in brain function observed in the present pilot investigation reflect habituation to the stimuli pre- and post-training. However, this is unlikely, as we have previously published data with this paradigm that included a control group and failed to show habituated brain activation (see Haase et al., 2014). Last, the literature suggests training can alter perception/confidence in interoceptive performance without actually affecting performance itself (Khalsa et al., 2008). As such, the use of self-report measures (e.g., MAIA, TAS, FFMQ), in the absence of any sensitive behavioral changes in interoception, make it hard to know whether the athletes just believe they are better as a result of mPEAK training. Interoception – similar to emotion – can be conceptualized from a (a) phenomenological perspective, i.e., how something feels, (b) an operant perspective, how something affects measurable behavior, and (c) from a physiological perspective, i.e., what biological measures are associated with it. In this report we present evidence for (a) and (c) but not for (b). There are several possibilities that we will evaluate in the future, which focus on (1) sensitivity of the behavioral assessments of interoception, (2) state-dependency (i.e., intra-individual variability), and (3) cross-modal interoceptive sensitivity, e.g., sensitivity to breathing load as it relates to heartbeat detection.

## Conflict of Interest Statement

The authors declare that the research was conducted in the absence of any commercial or financial relationships that could be construed as a potential conflict of interest.
